# Coalition of Biology and Chemistry for Ameliorating Antimicrobial Drug Discovery

**DOI:** 10.3389/fmicb.2017.00734

**Published:** 2017-05-04

**Authors:** Dipesh Dhakal, Jae Kyung Sohng

**Affiliations:** ^1^Department of Life Science and Biochemical Engineering, Sun Moon UniversityAsan-si, South Korea; ^2^Department of BT-Convergent Pharmaceutical Engineering, Sun Moon UniversityAsan-si, South Korea

**Keywords:** antimicrobial drugs, biological engineering, synthetic biology, chemical synthesis methods, metabolic engineering

Natural products (NPs) are superior starting point for the major antimicrobials used in clinical trials (Newman and Cragg, 2012; Butler et al., 2014). Such antimicrobial NPs can be obtained from different microorganisms (Polpass and Jebakumar, 2013) and plants (Atanasov et al., 2015). They can be broadly classified as (i) native NPs, (ii) derivatives of NPs, or (iii) synthetic products based on structures of NPs (Demain and Sanchez, 2009). While NPs exhibit a wide range of pharmacophores and a high degree of stereochemistry (Harvey et al., 2015), novel NPs with better activities still need to be developed. Versatile biological knowledge based synthetic-biology approaches, system-biology guided metabolic engineering techniques, enzymatic modifications, and synthetic chemistry methods can be utilized to maximize the benefit of NPs from the source organism (Dhakal et al., 2015, 2016). Thus, the optimum application of NPs can only be improved with considerable effort based on precise screening, higher production, and desirable structural diversification (Dhakal and Sohng, 2015).

## Engineering of biological systems

The antibacterial compounds can be produced by microorganisms to higher multicellular organisms as plants and animals from marine and terrestrial sources (Berdy, 2005; Hayashi et al., 2013). Fundamentally, a stable, well-behaved chassis (host cell) is essential for the production of desirable molecules at a significant level (Keasling, 2012). For native source like plants, the yield of target molecule is lower, that too comes through hectic separation among multitude of other structurally similar compounds (Chemler and Koffas, 2008). Hence, easily manipulable biological systems (production hosts) are attractive platforms for the large-scale production of major NPs. These biological systems can be programmed to produce chemicals of interest with higher precision and cost effectiveness (Stephanopoulos and Vallino, 1991). The synergistic implementation of synthetic biological tools and system biology assisted metabolic engineering approaches can be fundamental for harnessing maximum utility from the biological systems (Stephanopoulos, 2012; Nielsen et al., 2014). Synthetic biology deals more precisely with the design and construction of new biological systems (e.g., genetic control systems, metabolic pathways, cells) that do not exist in nature. Metabolic engineering approaches concentrate more on the development of methods and concepts for analysis of metabolic networks or pathways, typically for finding targets to re-engineer cell factories (Nielsen et al., 2014). Whilst engineered microorganisms are used to produce target compounds, synthetic biology, and metabolic engineering overlap at the level of metabolic pathway construction, enabling biocatalyst engineering, and maximizing cellular productivity (Nielsen and Moon, 2013).

**Figure 1:**
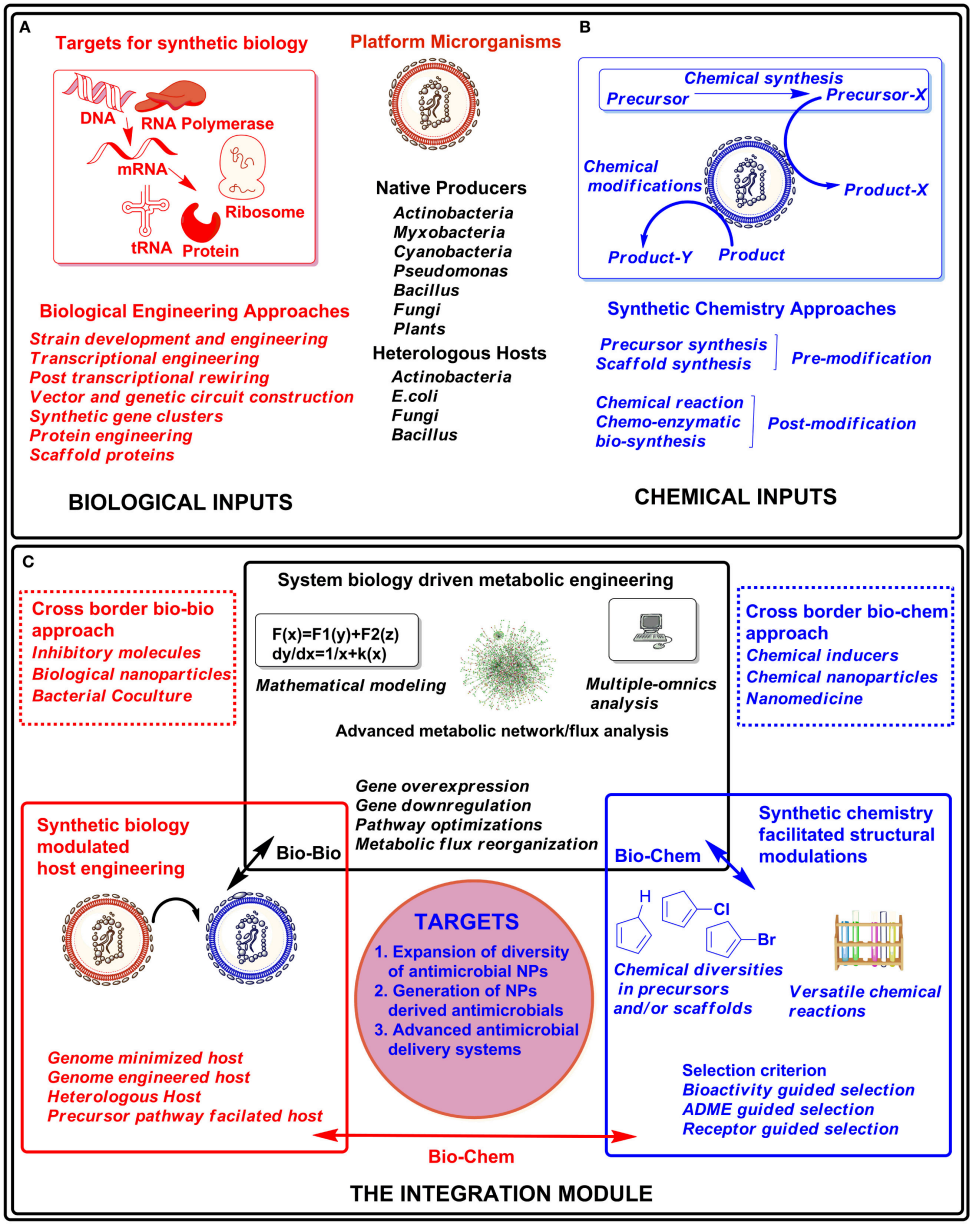
**Graphical summary of the integrated approach for drug discovery and development**. The biological engineering approaches **(A)** and chemical inputs through synthetic chemistry **(B)** can be applied to producer hosts (native or heterologous) or their products from those hosts. These biological engineering approaches and combinatorial chemical diversity can be integrated in modular form **(C)** for developing novel or effective drug molecules.

Some NPs are not amenable for production at a significant level or cryptic in the native host, or the hosts are genetically intractable. “Genome mining” approach has enabled the computation of mined genetic data and the connection to particular NPs, even if they are cryptic or produced in insignificant titers (Ziemert et al., 2016). Thus, native hosts, genetically tractable alternative hosts or suitable heterologous hosts, can be used as a platform for system-level metabolic engineering approaches (Luo et al., 2015; Dhakal et al., 2016). Some of the key foundations (Figure 1) are briefly summarized below.

### Strain development and engineering

The design of a metabolic pathway needs to be well-supported with an appropriate host, because a suitable host is only able to manifest the computational design or recombineered genetic circuit in the form of a product (Medema et al., 2011; Keasling, 2012). The classical strain engineering techniques of mutate and screen (Adrio and Demain, 2006) have advanced to such extent that we are now entering to an synthetic biology approaches for developing efficient native/heterologous hosts (Luo et al., 2015). *Actinomycetes* species as *S. coelicolor* M1152 (Gomez-Escribano and Bibb, 2011) and *S. avermitilis* SUKA (Komatsu et al., 2013) have been successfully employed for heterologous production of different antimicrobials. Recently, the heterologous expression systems have been extended in other organisms as *Bacillus subtilis* (Li et al., 2015), *Escherichia coli* (Ross et al., 2014), and Aspergillus niger (Anyaogu and Mortensen, 2015). Various rapid, high throughput and automated multiplex genome engineering techniques are providing advanced avenues for making it feasible to generate compound specific superhosts (Nyerges et al., 2016; Wang et al., 2016). However, the major constraint is finding the appropriate host for particular NPs, due to host specific discrepancy in metabolism particularly in terms of flux balance between primary metabolites (precursors) and secondary metabolites (target molecules).

### Metabolic pathway design and engineering

The discrepancy of metabolism in host can be reconfigured by design of optimal pathways leading to production of natural-noninherent or non-natural-noninherent molecules. Further introduction of enzymes with superior catalytic activity or rationally engineered by domain swapping, mutagenesis etc. can establish a perfect metabolic pathway and overcome the problem of rate limiting cascades (Lee et al., 2012). These *in silico* optimizations of major metabolic pathways or ancillary precursor supply pathways have been instrumental in the efficient production different molecules with antimicrobial values (Medema et al., 2012). But, the major drawback for most efficient metabolic pathway designs and redesigns for particular host is prerequisite information of its genome and expression profiles.

### Systematic modulation of metabolic pathways

In absence of complete information of genome or expression levels as well, engineering of an appropriate host and metabolic circuit can be assisted by the systematic modulation of the pathways. The controlled tuning based on regulated expression from promoters (Alper et al., 2005; Zhang et al., 2010) or the ribosome binding site (RBS) (Salis et al., 2009) is most commonly used for this purpose. Vicinity engineering by protein fusion (Yu et al., 2015) or the use of scaffolds of DNA (Conrado et al., 2012), RNA (Delebecque et al., 2011), and protein (Dueber et al., 2009) provides an amenable platform for increasing the rate of enzyme catalysis. Meanwhile, the complete metabolic pathway can be reorganized by generating an artificial pathway for smooth flow over the metabolic nodes, previously identified as bottlenecks (Luo et al., 2015). This tweaking of metabolic pathway assists in transferring the complex metabolic pathways in native/heterologous producer strain and thereof cost-effective production (Galanie et al., 2015). However, these type of systematic modulation are not easy to design and implement. There is lack of universality in these approaches depending on the metabolic genuinty of the host strain, which demands for numerous hits and trials, cost, and time.

## Rational contribution from synthetic chemistry

In case of un-culturable organisms or un-clonable pathways, total synthesis can be valid approach for accessing designed analogs of target NPs. Moreover, by combining the synthetic chemistry and biosynthetic approaches, the access to libraries of synthetically intractable analogs has been permitted (Goss et al., 2012). The methods for *in situ* cultivations in artificial iChip devices that mimic their natural environment enabled isolation of a new antibiotics teixobactin, without detectable resistance (Ling et al., 2015). The exploitation of “not-yet cultured” biodiversity by such approaches can lead to the discovery of more novel effective molecules (Kolter and van Wezel, 2016). The target based screening of NPs has been a popular technique for identifying effective NPs by phenotypic or genotypic assays (Wang et al., 2013; Lee et al., 2016). The exploration of new environmental settings including the study of human flora suggests a new direction in the isolation of previously unpredicted NPs (Zipperer et al., 2016). Thus the rational contribution of the synthetic chemistry to biology can therefore undoubtedly result in the expeditious generation of libraries of novel compounds with better activities (Figure 1).

### Pre-modification or supplementation approach

The chemical synthesis of some precursors or scaffolds can be used to generate novel analogs. Basically, these can be: (1) precursor directed biosynthesis (Harvey et al., 2012) or (2) mutasynthesis (Kennedy, 2008). Precursor directed biosynthesis utilizes natural flexibility of the biosynthetic pathway toward accepting the chemically synthesized precursor analogs (Harvey et al., 2012). In mutasynthesis approach, the native biosynthetic enzymes are modified and an unnatural chemically synthesized building block is introduced to the engineered system. The host strain is engineered to limit the competitive pathways by the inactivation/exchange/modification of key enzymes to yield the maximum production of the desired products (Kirschning et al., 2007; Koryakina et al., 2016). Versatile antimicrobial compound classes have been generated by these approaches (Weissman, 2007; Kennedy, 2008). However, the stero/regio- specificity of the biosynthetic enzymes limit the abundant diversity, which can be amended by making substantial change in domain architecture of key biosynthetic enzymes.

### Post-modification or complementation approach

To combat the influence of biosynthetic enzyme over chemical scaffolds, direct intervention of synthetic chemistry has been adopted as: (1) chemical transformation or (2) chemoenzymatic biosynthesis. In the first approach, the biological product is processed through single/multiple chemical reaction and decorated with the desired chemical scaffold. In latter approach, the microbial product is processed through iterative modes of biosynthetic mechanism along with chemical reactions, thus multiplexing the parent compounds with novel functionalities and delivering chemical libraries as new leads (Yan et al., 2013; Kim et al., 2015). The diversified chemical scaffolds were synthetically prepared and recruited at different biosynthetic stages of antimycin (ATN) biosynthesis in *Streptomyces* sp. NRRL 2,288 to generate library of ~380 ATN-like compounds(Yan et al., 2013). Thus, these combinations of synthetic chemistry and biosynthetic engineering can be very promising approaches for generating diversified classes of antimicrobial compounds (Winn et al., 2016).

## The cross border interactome schema

The integration and interaction of synthetic chemistry with biological processes has definitely broadened the scope of the development of antimicrobial agents, such as:

### Nanomedicine

The combination/conjugation of NPs with the chemically or biologically generated nanoparticles has proven to be very popular (Singh et al., 2016). Nanoparticles are used for targeted drug delivery (Sun et al., 2014), enhancing the biological activities (Gurunathan et al., 2014), or enhancing the bioavailability (Xie et al., 2011). The toxicity and safety of such metallic nanoparticles for the clinical use is not well-studied. However, antimicrobial peptides (AMPs) with potent antibacterial activity are taken as potential drug candidate for treatment of various bacterial infections(Meneguetti et al., 2016). The structurally nanoengineered antimicrobial peptide polymers (SNAPPs) inspired by the naturally occurring AMP, are the best example of a biological concept facilitating the development of chemical synthesis based super-drugs (Lam et al., 2016).

### Perturbation of signaling factors

The pathogenic bacteria frequently release small molecules commonly called as virulence factors. The study of such virulence factor or other signaling molecules can assist in understanding bases of pathogenicity and designing inhibitor molecules (Anthouard and DiRita, 2015). These inhibitor molecules can be either derived from suitable strain or chemically synthesized to combat the growth of pathogenic bacteria. It has been observed that there is the elicitation of novel NPs by cross-talk of some signaling molecules with other microorganisms in a co-culture (Goers et al., 2014) or the fermentation with chemical inducers (Yamazaki et al., 2015). The major limitation in these approaches is that most of them are solely based on hits and trials, nevertheless these biological-biological or chemical-biological interactome have proven contribution in production of known molecules and even expression of various cryptic molecules (Bertrand et al., 2014; Pimentel-Elardo et al., 2015).

## Future perspective

The chemistry directed evolution in NPs discovery has provided diversified bioactive molecules or chemical modulators. But the traditional chemical processes are limited in diversity generated through “one-synthesis/one-scaffold” approach (Hong, 2011), whereas effective antimicrobial NPs demands remarkable structural complexity (Luo et al., 2015). Moreover such processes are expensive requiring multiple reagents, equipment etc., and often leads to production of hazardous chemicals (Jha et al., 2015). In case of microbial cell factories (native, engineered, or heterologous) the necessary titer, yield, and productivity for industrial application is on demand, but generally difficult to achieve (Porro et al., 2014). The major constraint is appropriate flux balance for diverting primary metabolites to target molecules. Hence revolutionizing the drug discovery and development process by integrating both biological and chemical techniques to complement each other is long overdue. The chemically optimized precursors/scaffolds can be processed through bio-engineered super-hosts to generate antimicrobial compounds in combination and permutation modes. Furthermore, studies on novel drug delivery methods utilizing integrated approach of biology and chemistry have potential to broaden the utility and efficacy of these antimicrobial molecules (Abed et al., 2015). In summary, this integrative modular approach (Figure 1) can provide repositories of antimicrobials for eradication of different infections.

## Author contributions

DD and JS made substantial, direct and intellectual contribution to the work, and approved it for publication with full consent.

### Conflict of interest statement

The authors declare that the research was conducted in the absence of any commercial or financial relationships that could be construed as a potential conflict of interest.
